# Making a Realist Turn: Applying a Critical Realist Translational Social Epidemiology Methodology to the Design and Evaluation of Complex Integrated Care Interventions

**DOI:** 10.5334/ijic.4725

**Published:** 2019-07-25

**Authors:** John Eastwood

**Affiliations:** 1 Healthy Homes and Neighbourhoods Integrated Care Initiative, AU; 2 Community Paediatrics, Child and Family Clinical Services, Community Health, AU; 3 Clinical Services Integration and Population Health, AU; 4 Sydney Institute for Women, Children and their Families, Sydney Local Health District, Camperdown, NSW, AU

**Keywords:** Critical Realism, Translational Social Epidemiology, Integrated Care, Complex Interventions, Design, Evaluation

*“Building on the SDG goal of achieving universal health coverage, WHO has developed a global strategy and Framework for people-centered and integrated health services. … The framework proposes five interdependent strategies for health services to become more integrated and people-centred. They are: (1) empowering and engaging people and communities; (2) strengthening governance and accountability; (3) reorienting the model of care; (4) coordinating services within and across sectors; and (5) creating an enabling environment”[1]*.

The challenge of achieving people-centred integrated health [and social] services requires the scientific study of interventions and improvements that can achieve the above mutually supporting strategies. Both implementation and improvement science have contributions to make here, but we content that a critical realist methodology will enable a deeper understanding of “how and why things are”, and “what will work for whom in what circumstances”.

Implementation science concerns itself with scientific enquiry into matters related to implementation of policies, programmes or individual practices. Much of the current focus is on the translation and implementation of “evidence-based” interventions that were shown to have been efficacious in a controlled setting. The goal of improvement science is to determine which improvement strategies work to assure effective and safe patient care. The intent of both implementation and improvement science in health and social care is to understand how and why interventions work with “real people” in the “real” world, such as with *people-centered integrated health [and social] services*.

Health and social care is conducted, however, within complex systems where the various human actors (with agency) interact with existing, and changing, social and organisational arrangements or “structures”. Interventions that seek to change observed phenomenon, such as disease patterns, will be influenced by these existing conditions. Consequently it is not sufficient to simply assess a change in outcomes or to describe the intervention process. It is instead necessary to diagnose the extant conditions, and then to design, apply and evaluate the prescribed interventions in those “real world” conditions. Here, and in the first four papers of this supplement, we will advance that a critical realist research, design and evaluation approach has merit and should be conducted in partnership with those who will be affected.

For physicians, and other health practitioners, the process will be very familiar as it involves the same modes of study and reasoning undertaken in a complex clinical consultation. In drawing this analogy we highlight the importance of: patient history, context, objective findings, theory building, differential diagnosis or best explanation, evidence-based treatment, monitoring, and re-evaluation of assumptions. The monitoring and re-evaluation is necessary because of patient behaviour, influence of external conditions, fallibility of the diagnosis process, and variability in treatment effectiveness.

The above clinical and system analyses both require consideration of history, structure, culture, relationships, and human reasoning (agency). They both also assess the world in terms of biological, psychological, social, and economic domains. In addition to the observed, or empirical, they also both seek to understand and explain the hidden mechanisms using an “inference to the best explanation” form of reasoning. Finally both will most likely accept the possibility of fallibility, and that their reasoning processes are influenced by their own training, experience, values and beliefs. In taking the above approach, clinicians and system practitioners are arguably both using a critical realist philosophy of science, and the related abductive and retroductive forms of reasoning.

The Philosophy of Science, Critical Realism, is “realist” in that it holds that objects in the world, and in particular social objects, exist whether the observer or researcher is able to know them or not; and it is “critical” in seeing knowledge of those objects as always fallible because any attempts at describing them needs to take account of the transitive nature of knowledge. Understanding the world in terms of these intransitive and transitive dimensions has proven especially helpful when applied to the study health and social systems. The critical realist approach to considering reality as stratified, for example in to biological, psychological, social, and economic domains, makes it especially useful for the study of social and organisational systems and for the discipline of translational social epidemiology as it might be applied to integrated care. While focusing here on the discipline of social epidemiology, we acknowledge that similar methods may be used for “organisational research”.

Social epidemiology has developed over the last twenty years a sophisticated approach to the study of health and social systems. To date social epidemiology has been dominated by empirical descriptive studies that draw attention, for example, to the impact of service accessibility, acceptability, availability, and cost, on health and social outcomes. We have previously drawn attention to the challenge for social epidemiology to make a turn from its descriptive empirical “humean” roots toward an applied realist translational discipline, thus using its tools to move from explaining health and social phenomenon, toward designing, implementing and evaluating complex health and social interventions. Such a turn calls for a transdisciplinary approach that moves from studying historical and current organisational and social structures and human behaviours, explaining the observed phenomenon through to the explication of theoretical propositions, and finally designing and evaluating interventions that are most likely to be complex in nature [2].

Muntaner [3] argued for the use of a realist methodology that seeks to generate social interventions in partnership with the affected populations. In making this argument Muntaner was challenging social epidemiologists to move from the study of causal mechanisms (i.e. realist causal theory) toward the applied development of implementation and programme theories [4]. We seek to contribute towards this challenge, and describe a realist translational social epidemiology methodology for the translation of empirically ‘data derived’ causal middle-range theories of social mechanisms, into social programmes (with programme theories). Those theoretical propositions can be operationalised and studied in concrete situations using theory driven approaches.

O’Campo and Dunn [5] have recently observed that “if social epidemiology continues in its current path, we are likely to see a continued growth in empirical studies demonstrating the existence of a variety of different health inequalities, with relatively little contribution to studies that characterise and inform solutions to those inequalities”. We contend that the identification of solutions requires that we change approach from identifying associations or regularities in empirical data to the identification of the causal explanatory mechanisms, and consequently the application of programme interventions that impact on those causal mechanisms.

In response to this challenge we undertook a critical realist social epidemiology programme of research that sought to build a “Theory of Neighbourhood, Stress, Depression and the Developmental Origins of Health and Disease (DoHD)” using maternal postnatal depression as a case-study [2,6]. A Multi-level concurrent triangulated mixed method study was used to build a realist middle-range theory using an *Explanatory Theory Building Method* [2].

We will described in the accompanying collection of papers a realist translational social epidemiology programme of research that will use the meta-theory of critical realism to concretise and contextualise the previously described critical realist theory of neighbourhood context, stress, depression and the developmental origins of health and disease. We will situate these studies in the socially disadvantaged regions of Sydney where the local child and family inter-agencies are collaborating to design and implement new programme interventions based on our earlier studies of perinatal, child, youth and family outcomes.

In preparing this methodology we transverse several areas of epistemological and methodological controversy including: critical *versus* scientific realism; MCO, CMO and CIMO forms of realist propositions; causal, programme and implementation theory; Theory of Change *versus* Realist Evaluation; and the methodological place of statistical structural modelling within a critical realist epistemology.

Central to the methodology is the mixed method study of the extant condition including the history. This may not be immediately obvious from this body of work as our study of the base-conditions was published earlier. We argue that this step is essential in all situations in order to develop a thorough understanding of the pre-existing and current context within which evidence-informed practices will assist program design and implementation, rather than dictate them.

Much of the current theory driven and realist evaluation literature begins with existing interventions. The first task in those situations is to identify the underlying programme theory. In preparing the programme of work described here, we were faced with the translation of causal theory to programme and implementation theory. We have proposed as a first step in this methodology the formal translation of the middle-range ‘causal’ theory into a middle-range ‘programme theory’ followed by an intervention design process based on Theory of Change approaches.

The work of Denyer and co-authors [7] is helpful here in making explicit the requirement for a design step in the realist evaluation cycle and Keller and colleagues [8] introduce CIMO logic as a useful step in the translation process. It is important to acknowledge here the important contribution that shared visions and collective planning will make to the development of successful Theory of Change. Consumer and practitioner input to the design and evaluation of interventions is critical to their success. We will include two sequential papers that describe the application of the methodology to design 1) an integrated care intervention for vulnerable families and 2) the funded integrated care intervention Healthy Homes and Neighbourhoods.

Also included is a protocol that will describe our application of critical realist meta-theory to the UK Medical Research Council (MRC) Framework for evaluating complex health interventions. This Framework has four components, namely 1) development, 2) feasibility/piloting, 3) evaluation and 4) implementation. We adapted the Framework to include: critical realist, theory driven, and continuous improvement approaches. The original MRC Framework, as is common in the field of implementation science, focused only on activities and outcomes. The incorporation of a critical realist methodology added a focus on history, context and mechanisms. The modified framework used a multilevel approach that used critical realist mixed-method research to examine not only outcomes, but also *what is working for whom and why*.

At the recent International Conference on Integrated Care in Utrecht we called for the establishment of an IFIC special interest group (SIG) for “Realist Research, Design and Evaluation”. The SIG aims to bring together health, education and social care practitioners and researchers who are interested realist research methods for integrated care. As a group we will discuss service, policy and system approaches and collaborate on Integrated Care research and development projects, including grant proposals.

Our first steps will include: establishing a global network of interested parties; define the role of realist research methods within integrated health and social care; develop position paper; develop a framework for the application of realist research methods within the study of integrated service, policy and system approaches for integrated care; collaborate on research and grant proposals; develop and promote appropriate realist research methods; share knowledge, successes, lessons-learnt, and current models of care; establish a community website, and meet during the International Conferences on Integrated Care. There are challenges in evaluating outcomes and the complex interventions required for integration of care, and thus the required methodological approaches will be a key issue for early discussion within the SIG.
